# A Rare Case of Primary Extra-Nodal Marginal Zone Lymphoma of Mucosa-Associated Lymphoid Tissue (MALT) in the Rectum

**DOI:** 10.7759/cureus.49447

**Published:** 2023-11-26

**Authors:** Ali Tariq Alvi, Murali Shankar

**Affiliations:** 1 Internal Medicine, HCA Florida Westside Hospital, Plantation, USA; 2 Internal Medicine, HCA Florida Northwest Hospital, Margate, USA

**Keywords:** rectal mass, rectal bleeding, rectum, malt, lymphoma

## Abstract

Mucosa-associated lymphoid tissue (MALT) is a unique clinical condition that can manifest in different anatomic locations. In the gastrointestinal tract, it is typically seen in the stomach but is less commonly found in other sites. There have been a few cases in the literature in which primary MALT lymphoma is found in the rectum. We describe a case of a 63-year-old male who presented with rectal pain and bleeding. Colonoscopy revealed a rectal mass, which was excised with a trans-anal approach. Histopathological examination of the biopsy specimen was significant for MALT lymphoma. Therefore, the patient underwent radiation therapy followed by repeat colonoscopies to monitor disease recurrence.

## Introduction

Extra-nodal marginal zone lymphomas are indolent non-Hodgkin lymphomas that develop from memory B cells in post-germinal centers and differentiate into plasma cells and marginal zone cells [1]. These are considered low-grade and less common than diffuse large B cell lymphomas, accounting for almost 38% of cases of all primary gastric lymphomas [2,3]. The incidence of mucosa-associated lymphoid tissue (MALT) lymphomas has been reported to be highest in the fifth and sixth decades of life [4]. 

*Helicobacter pylori* is the most common infectious agent associated with malignancies, accounting for 5.5% of worldwide cancers. It is commonly associated with the development of gastric MALT lymphomas [5]. The stomach is the most frequently associated site of MALT lymphomas, accounting for 35% of cases, followed by involvement of less common sites, including ocular adnexa, salivary glands, colon, lungs, and small intestine [6]. It is rare to observe a MALT lymphoma in the rectum, with only a few cases published in the literature. 

## Case presentation

**Figure 1:**
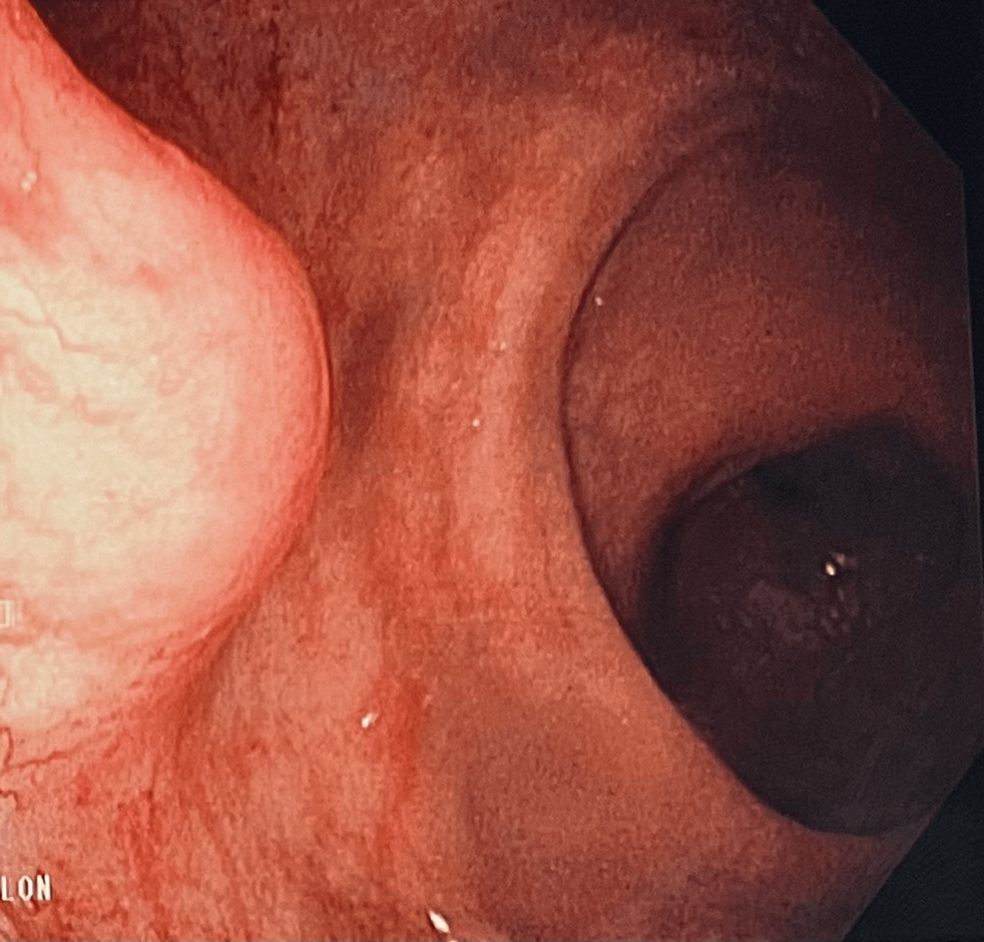
Routine colonoscopy demonstrating well-defined rectal mass on the lateral wall of the rectum

We describe a 63-year-old African-American male with a medical history of essential hypertension, hemorrhoids, colonic polyps, left ulnar nerve damage, and tobacco use disorder who presented with intermittent rectal pain and bleeding for the last few weeks. He had a family history of colon cancer in his brother. His last colonoscopy was four years ago, during which four polyps were removed. He denied any history of hematemesis, melena, dysphagia, odynophagia, non-steroidal anti-inflammatory drug use, or recent weight loss. The fecal occult blood test was positive. A computed tomography (CT) scan of the abdomen and pelvis was performed, and it was unremarkable. During the colonoscopy, a well-defined submucosal rectal mass was observed on the lateral aspect of the rectal wall, measuring approximately 3.4 cm x 1.7 cm x 1.5 cm in dimensions (Figure 1).

Therefore, it was excised with a trans-anal approach. Histopathological examination of the biopsy specimen demonstrated highly atypical B-cell proliferation consistent with extra-nodal marginal zone lymphoma involving rectal mucosa and submucosa. Immunohistochemical stains showed that lymphoid cells were diffusely positive for CD45, CD20, and Pax5 and were negative for CD5, BCL6, CD43, and CD10.

**Figure 2:**
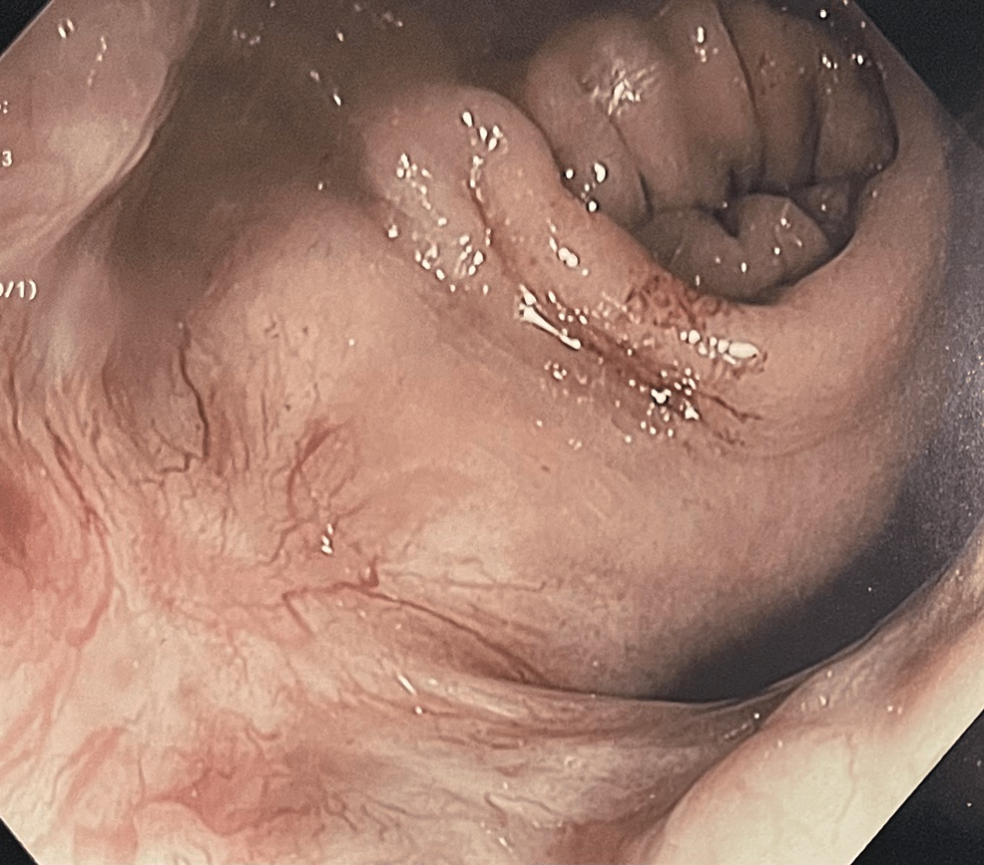
Colonoscopy demonstrating evidence of a previous mucosal scar at the site of resection with telangiectasia suggestive of radiation proctitis

Therefore, the patient was referred to radiation oncology for external beam radiation therapy. It was followed by colonoscopies every year, with no signs of disease recurrence thus far (Figure 2).

## Discussion

The role of *H. pylori* infection is significant in developing gastric MALT lymphomas, as the lymphoid tissue in gastric mucosa commonly arises due to chronic *H. pylori* infection [7,8]. The source and pathogenesis of MALT lymphomas of the rectum remains unclear. Some of these patients have been reported to have *H. pylori* infections of the stomach. At the same time, a few of them were also found to have autoimmune conditions like Hashimoto’s thyroiditis or Sjogren’s syndrome [9-11]. The common presenting features in patients with colon and rectal MALT lymphoma include constipation, diarrhea, abdominal discomfort, or positive fecal occult blood test [6]. The patient described in this case presented with intermittent rectal pain and bleeding.

MALT lymphomas in the colorectal region may present with diverse endoscopic characteristics, including raised, flat, polypoid, or semi-pedunculated tumors, which often exhibit a nodular, granular, or smooth surface [6,12,13]. Tumor size can also differ, with most falling within the range of 15-30 mm in dimension [6]. According to a study that evaluated multiple cases of primary rectal MALT lymphomas, polypoid lesions were found to be 10 times more prevalent than ulcerative lesions, with concurrent involvement of colon or cecum also observed in 20% of cases [13]. The patient described in this case also had a polypoid lesion with no involvement of the cecum or colon. 

The treatment options for MALT lymphomas of the rectum encompass *H. pylori* eradication, surgical resection, various chemotherapeutic medications, and endoscopic mucosal resection [8]. Some studies have documented regression of rectal MALT lymphoma after initiation of triple therapy against *H. pylori*, even in patients who tested negative for this infection [14,15]. However, there are still no clear guidelines on approaching this condition clinically. That is why we decided to excise the tumor with a trans-anal approach followed by radiation therapy. 

## Conclusions

MALT lymphomas are typically associated with the upper gastrointestinal tract but are infrequently seen in the rectum. This case report highlights the significance of keeping the MALT lymphoma in the differential diagnosis of patients having rectal lesions observed on colonoscopy. Additionally, while determining the appropriate treatment approach for MALT lymphomas in the rectum, the patient-specific factors, including the extent of disease, age, and underlying comorbidities, should also be considered. Further research is necessary to explore various aspects before definitive treatment strategies can be established for managing patients with this condition.
